# Understanding competing risks: a simulation point of view

**DOI:** 10.1186/1471-2288-11-86

**Published:** 2011-06-03

**Authors:** Arthur Allignol, Martin Schumacher, Christoph Wanner, Christiane Drechsler, Jan Beyersmann

**Affiliations:** 1 Freiburg Center for Data Analysis and Modeling, University of Freiburg, Germany; 2 Institute of Medical Biometry and Medical Informatics, University Medical Center Freiburg, Germany; 3 Department of Medicine 1, Division of Nephrology, University of Wuerzburg, Germany

## Abstract

**Background:**

Competing risks methodology allows for an event-specific analysis of the single components of composite time-to-event endpoints. A key feature of competing risks is that there are as many hazards as there are competing risks. This is not always well accounted for in the applied literature.

**Methods:**

We advocate a simulation point of view for understanding competing risks. The hazards are envisaged as momentary event forces. They jointly determine the event time. Their relative magnitude determines the event type. 'Empirical simulations' using data from a recent study on cardiovascular events in diabetes patients illustrate subsequent interpretation. The method avoids concerns on identifiability and plausibility known from the latent failure time approach.

**Results:**

The 'empirical simulations' served as a proof of concept. Additionally manipulating baseline hazards and treatment effects illustrated both scenarios that require greater care for interpretation and how the simulation point of view aids the interpretation. The simulation algorithm applied to real data also provides for a general tool for study planning.

**Conclusions:**

There are as many hazards as there are competing risks. All of them should be analysed. This includes estimation of baseline hazards. Study planning must equally account for these aspects.

## Background

The analysis of time-to-event data ('survival analysis') has evolved into a well established application of advanced statistical methodology in medicine. E.g., in the *New England Journal of Medicine*, survival methods have evolved from an occasionally used technique in the late 70s over moderate use in the late 80s into the leading statistical procedure by 2005 [1]. The archetypical application analyses time until death, but combined endpoints are also frequently considered. E.g., a recent literature review in clinical oncology [2] found a multitude of combined endpoints including, e.g., progression-free survival, distant metastasis-free survival, locoregional relapse-free survival, etc. The medical problems at hand will, as these endpoints exemplarily suggest, usually be more complex than can be addressed by the analysis of time until one potentially combined event type.

Competing risks techniques allow for a more specific analysis in that they consider time until occurrence of the combined endpoint *and *endpoint type, e.g., progression in contrast to death without prior progression. The relevance of competing risks in medical research is highlighted by methodological papers in various medical fields. We mention [3-5] as recent examples. A classical statistics textbook account has been given in the first edition of [6] in 1980, and a definite mathematical treatment based on counting processes is included in [7]. Excellent tutorial papers in the statistical literature are [8,9].

However, despite an obvious practical relevance and a firmly established methodological background, competing risks are not always well accounted for in published survival analyses in medical journals: E.g., another recent literature review [10] in clinical oncology found that 27 out of 125 included randomised controlled trials considered time to progression, but only 5 out of 125 studies accounted for such endpoint types being non-exclusive. A similar picture has been reported for studies on the effect of implantable cardioverter defibrillator on subsequent cardiac events [11].

The key to an adequate competing risks analysis is: There are as many hazards as there are competing risks. Unless all of these hazards, which are often called cause-specific hazards, have been analysed, the analysis will remain incomplete. In particular, only a complete analysis will allow for predicting event probabilities.

The aim of this paper is to suggest an algorithmic or simulation point of view towards this key issue. The idea behind this point of view is that it gives a clear building plan of how the involved hazards generate competing risks data over the course of time. However, although the algorithm is mathematically well established [12], it is rarely used in practice [13]. We will use it as an operational device for understanding competing risks.

The remainder of the paper is organised as follows: The Methods Section introduces competing risks as arising from a multistate model, gives an algorithmic interpretation of the involved hazards and provides a description of the data example from a recent study on cardiovascular events in diabetes patients [14]. In this Section, we also explain 'empirical simulation', where one simulates based on the empirical study probabilities, and give an overview on simulation scenarios that will be considered. The Section closes with a brief summary of the difference between the present paper and the common latent failure time approach to competing risks. Results are reported in the Results Section. Our simulations are 'empirical simulations', and they work as a proof of concept: Interpreting a competing risks analysis from a simulation perspective can be viewed as a thought experiment. The actual simulations show that the simulation perspective works. Finally, a discussion and a conclusion are offered, with a focus on consequences for practical competing risks analyses, including graphical presentation and planning in the presence of competing events.

## Methods

### The competing risks multistate model

**Figure 1:**
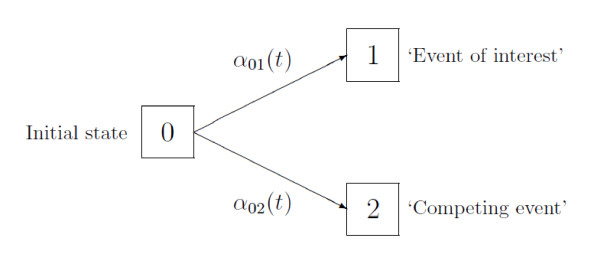
**Competing risks multistate model**. Competing risks multistate model with cause-specific hazards *α*_0*j *_(*t*), *j *= 1, 2.

Figure 1 depicts the multistate model of a competing risks process (*X_t_*)_*t*≥0 _with initial state 0 and two competing event states 1 and 2. *X_t _*denotes the state that an individual is in at time *t*. The restriction to two competing events is for ease of presentation only. Initially, every individual is in state 0 at time origin, i.e., *X*_0 _= 0. The individual stays in this state, i.e., *X_t _*= 0 until occurrence of any first event. Usually, there is one event of interest, modelled by transitions into state 1, and all other first event types may be subsumed into the competing event state 2.

The competing risks process moves out of the initial state 0 at the event time *T*. At time *T *, the process enters one of the competing event states 1 or 2. Hence, the state *X_T _*that an individual is in at time *T *denotes the type of the first event, often called cause of failure. *X_T _*equals either 1 or 2.

Key quantities in competing risks are the cause-specific hazards (CSHs) *α*_0*j *_(*t*), *j *= 1, 2,(1)

The dt notation in (1) is a short, but more intuitive form of the more common *α*_0*j*_(*t*) = lim_Δ*t*↘0 _P(*T *∈ [*t*, *t *+ Δ*t*), *X_T _*= *j *| *T *≥ *t*)/Δ*t*. The interpretation of (1) is that the CSH at time *t *times the length d*t *of a very (infinitesimally) small time interval equals the conditional probability of making an 0 → *j *transition within that very small time interval [*t*, *t *+ d*t*). The CSHs can be envisaged as forces of transition, which work along the arrows in Figure 1. Analogous to cumulative distribution functions, basic statistical inference addresses cumulative quantities. We therefore also write *A*_0*j *_(*t*) for the cumulative CSHs, *A*_0*j *_(*t*) = .

The CSHs are key, because, as seen below, the pertinent probabilities are deterministic functions of the CSHs, and because both nonparametric estimation and Cox-type regression modelling build on them. The fundamental nonparametric estimator is the Nelson-Aalen estimator of the cumulative CSHs

*j *= 1, 2, where the summation is over all observed event times *s *≤ *t*. Variance estimation and, properly standardised, asymptotic normality of  is discussed in detail in Chapter IV of [7]. The most important regression approach are Cox models for the CSHs. If, as is usual, we assume that covariates have different, i.e., cause-specific effects on the CSHs, the partial likelihood factorises such that a Cox model for *α*_01_(*t*) may be fitted by additionally censoring type 2 events, and vice versa for a Cox model for *α*_02_(*t*), see, e.g., Chapter VII of [7]. The R [15] packages mvna[16] and survival[17] provide for convenient computation of the Nelson-Aalen estimator and the Cox model, respectively.

The *α*_0*j*_'s sum up to the all-cause hazard *α*_0·_(*t*)d*t *= *P *(*T *∈ d*t *| *T *≥ *t*) with cumulative all-cause hazard *A*_0·_(*t*). The survival function of *T *is therefore a function of both *α*_0*j*_'s, . As usual, *P *(*T *>*t*) is estimated using the Kaplan-Meier estimator, which is a deterministic function of the cause-specific Nelson-Aalen estimators.

The so-called cumulative incidence functions (CIFs) are the expected proportion of individuals experiencing a certain competing event over the course of time,(2)

*j *= 1, 2. The interpretation of the right hand side of (2) is that the CIF at time *t *equals the integral over all 'probabilities' to make an 0 → *j *transition precisely at time *u*. For this, an individual has to stay in state 0 until just before time *u*, which is reflected by the term *P *(*T *>*u*-). Conditional on being in state 0 just before time *u*, an 0 → *j *transition at time *u *is reflected by the term *α*_0*j *_(*u*) d*u*.

One consequence is that the CIFs are involved functions of all CSHs via *P *(*T *>*u*-). The CIFs *P *(*T *≤ *t*, *X_T _*= 1) and *P *(*T *≤ *t*, *X_T _*= 2) add up to the all-cause distribution function 1 - *P *(*T *>*t*). The Aalen-Johansen estimators of the CIFs can be obtained from (2) by substituting *P *(*T *>*u*-) with the Kaplan-Meier estimator and *α*_0*j *_(*u*) d*u *with the increment of the cause-specific Nelson-Aalen estimator. A detailed discussion of the Aalen-Johansen estimator is in Chapter IV of [7]; variance estimation is assessed in [18]. In R, survival is typically used for estimating *P *(*T *>*t*). The Aalen-Johansen estimator may be computed using etm[19], and prediction of the CIFs based on Cox models for the CSHs is implemented in mstate[20].

It is crucial to any competing risks analysis that both cause-specific hazards completely determine the stochastic behaviour of the competing risks process [7, Chapter II.6]. This is mirrored above by the CIFs and the all-cause distribution function being deterministic functions of all CSHs. However, these deterministic relationships are involved. Therefore, we will pursue an algorithmic interpretation of the CSHs below.

### Algorithmic interpretation of the cause-specific hazards

Thinking of the CSHs as momentary forces of transition which move along the arrows in Figure 1 suggests that competing risks data are generated over the course of time as follows:

1. The event time *T *is generated with distribution function 1 - *P *(*T *>*t*), i.e., with hazard *α*_01_(*t*) + *α*_02_(*t*) = *α*_0·_(*t*).

2. At time *T *, event type *j *occurs with probability *α*_0*j *_(*T *)/*α*_0·_(*T *), *j *= 1, 2.

Using this algorithm for simulation studies in competing risks has been discussed in [13]. It is important to note, however, that the algorithm goes beyond the computational question of how to implement simulations. Rather, the algorithm reflects the probabilistic question of how to build a probability measure based on the CSHs. This aspect is discussed in detail by [12] in the more general context of multistate models which are realized as a nested series of competing risks experiments.

The algorithmic perspective of this paper then implies that the task of statistical inference is to detect the ingredients of the above algorithm. We illustrate this approach in the data example below. To this end, we note that the analysis of a combined endpoint is restricted to step 1 of the above algorithm. Here, the effect of a treatment, say, on both CSHs determines whether the occurrence of an event (of any type) is delayed or accelerated. In step 2, the *type *of an event again depends on the treatment effect on both CSHs. We illustrate below that interpretation is straightforward if the treatment effects on the CSHs work in opposite directions or if one CSHs remains unaffected. However, interpretation will become more challenging if there are unidirectional effects on both CSHs. We will find that, in general, it is also mandatory to consider cause-specific baseline hazards in the interpretation.

### The 4D study

The background of the 4D study was that statins are known to be protective with respect to cardiovascular events for persons with type 2 diabetes mellitus without kidney disease, but that a potential benefit of statins in patients receiving hemodialysis had until then not been assessed. Patients undergoing hemodialysis are at high risk for cardiovascular events. The 4D study was a prospective randomised controlled trial evaluating the effect of lipid lowering with atorvastatin in 1255 diabetic patients receiving hemodialysis. Patients with type 2 diabetes mellitus, age 18-80 years, and on hemodialysis for less than 2 years were enrolled between March 1998 and October 2002. Patients were randomly assigned to double-blinded treatment with either atorvastatin (619 patients) or placebo (636 patients) and were followed until death, loss to follow-up, or end of the study in March 2004.

The 4D study was planned [21] and analysed [14] for an event of interest in the presence of competing risks. The event of interest was defined as a composite of death from cardiac causes, stroke and non-fatal myocardial infarction, whichever occurred first. The other competing event was death from other causes. Wanner et al. reported a CSH ratio of 0.92 (95%-confidence interval [0.77, 1.10]) for the event of interest. There was essentially no effect on the competing CSH.

**Figure 2:**
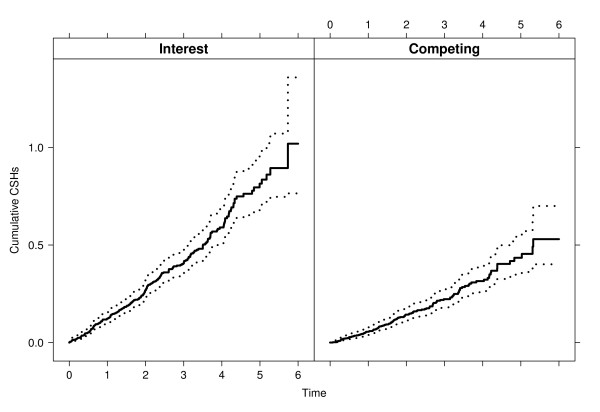
**Nelson-Aalen estimators of the cumulative CSHs**. Nelson-Aalen estimators (solid lines) of the cumulative CSHs with log-transformed 95% confidence intervals in the placebo group.

The simulation study below will use the data of the placebo group. In this group, 243 (38.2% of 636) events of interest and 129 (20.3%) competing events were observed. There were 264 (41.5%) censored patients. The Nelson-Aalen estimators  for the event of interest and  for the competing event are displayed in Figure 2.

### 'Empirical simulation'

Simulations were based on the empirical probabilities defined by . To avoid the issue of model misspecification, which is outside the scope of the present investigations, 'ideal' Cox models were used for generating data in the treatment group as explained below. Event times and event types were generated following the two-step algorithm described earlier.

To be specific, event times in the placebo group were drawn from the distribution defined by the Kaplan-Meier estimator derived from . Here, a practical complication arose in that the Kaplan-Meier estimator only spent about 76% of the probability mass. I.e., the Kaplan-Meier curve did not drop down to 0 because of right-censoring, which is a common phenomenon in clinical trials. This was handled by putting point mass beyond the largest observed time; corresponding realisations were always censored. In other words, on average about 100% - 76% = 24% of the simulated event times equalled some time that was larger than the largest observed time in the original data set. The corresponding observations were always censored with censoring times generated as explained below. Event types were generated by the binomial experiment of the two-step algorithm, substituting the CSHs by the increments of the cumulative CSHs. The algorithm was analogously applied, when transformed cumulative CSHs were used for the placebo group.

Data in the treatment group were generated based on 'ideal' Cox models. That is, CSH ratios exp(*β*_1_) for the event of interest and exp(*β*_2_) for the competing event were specified. If the original empirical baseline hazards were used, data were drawn based on the probabilities defined by . If transformations of  were used for generating placebo data, the CSH ratios acted on the transformed cause-specific baseline hazards. This reflects the situation that the true underlying baseline CSHs are *α*_01_(*t*) and *α*_02_(*t*) (or transformations thereof) in the control group, while the CSHs of the treatment group are exp(*β*_1_)*α*_01_(*t*) and exp(*β*_2_)*α*_02_(*t*) for the case of untransformed baseline hazards. Random censoring times were generated for all individuals based on the Kaplan-Meier estimator of the censoring survival function in the placebo group. Note that this estimator did spend 100% of the probability mass.

### Overview of simulation scenarios

The scenarios investigated in the next Section differed both with respect to the choice of *β*_1 _and *β*_2 _and in terms of the cumulative baseline hazards, i.e., the cumulative CSHs in the placebo group. The choice of the *β*'s broadly falls into three categories.

One category is characterised by *β*_2 _= 0, i.e., there is no effect on the competing CSH. A prime example are implantable cardioverter defibrillators [11], which display a beneficial effect on the CSH of sudden cardiac death but no effect on the CSH for death from other causes. The assumption of *β*_2 _= 0 straightforwardly implies the direction of the treatment effect on the CIF: If *β*_1 _< 0, the CIF of interest in the treatment group is always less than the one in placebo group. The relationship is reversed, if *β*_1 _> 0. This is intuitively understood thinking of the CSHs as momentary forces of transition, and it is reflected in step 2 of the simulation algorithm. E.g., if *β*_1 _< 0 and *β*_2 _= 0, the binomial event type 1 probabilities are reduced for the treatment group.

A second category is characterised by opposite treatment effects on the CSHs. This category straightforwardly implies the direction of the treatment effect on the CIF, too: The constellation *β*_1 _< 0 and *β*_2 _> 0 implies a smaller CIF of interest in the treatment group but also a larger competing CIF. These relations are reversed for *β*_1 _> 0 and *β*_2 _< 0. This is again intuitively implied by thinking of the CSHs as momentary forces of transition, and it is also reflected in step 2 of the simulation algorithm. E.g., if *β*_1 _< 0 and *β*_2 _> 0, the binomial event type 1 probabilities are reduced for the treatment group.

Finally, unidirectional treatment effects on the CSHs constitute the third category. Interestingly, the interpretation of unidirectional effects is straightforward when both competing events are fatal in the sense that a treatment with *β*_1 _< 0 and *β*_2 _< 0, say, is beneficial. But unidirectional effects also present the most challenging scenario in terms of understanding the resulting course of the CIFs. The interpretation for step 1 of the simulation algorithm is straightforward. If, e.g., *β*_1 _< 0 and *β*_2 _< 0, events of any type will happen later. The interpretational challenge becomes apparent in the second step of the algorithm: If, e.g., *β*_2 _<*β*_1 _< 0, the relative magnitude of the CSHs changes such that the binomial event of interest probabilities are increased. The interpretational difficulty is the *increase *of this probability, although *β*_1 _is *negative*. This constellation may result in an (eventually) increased CIF of interest.

All three categories are encountered in practice. Examples from clinical trials in hospital epidemiology are given in [22].

### Difference to the latent failure time model

Some readers may be more familiar with competing risks as arising from risk-specific latent times, say, *T*^(1) ^and *T*^(2)^. The connection to our multistate framework is *T *= min(*T*^(1)^, *T*^(2)^) with event type *X_T _*= 1, if *T*^(1) ^<*T*^(2)^, and *X_T _*= 2 otherwise. The latent failure time model imposes an additional structure, which has been heavily criticised mainly for three reasons. The dependence structure of *T*^(1) ^and *T*^(2) ^is, in general, not identifiable [23]. Since the latent times are unobservable, there is something hypothetical about them, which questions their plausibility [24]. Perhaps most importantly, it has been disputed whether the latent failure time point of view constitutes a fruitful approach to answer questions of the original subject matter [25].

Despite of this critique, latent times are the predominant approach for simulating competing risks data [13]. Assuming, for tractability, *T*^(1) ^and *T*^(2) ^to be independent with hazards equal to the cause-specific hazards *α*_01_(*t*) and *α*_02_(*t*), respectively, is computationally correct in that simulation based on this model yields the right data structure.

However, nothing is gained from assuming the additional latent structure, either. As a consequence, we will emphasise simulation and interpretation along the lines of the *Algorithmic interpretation of the cause-specific hazards *outlined earlier. This avoids the concerns on identifiability, plausibility and usefulness.

E.g., in the 4D study, the typical interpretation of the latent times would be that *T*^(1) ^is the time until death from cardiac causes, stroke or non-fatal myocardial infarction, while *T*^(2) ^is the time until death from other causes. Such an interpretation has given rise to debating whether, say, a patient may conceptually still die from other causes after having died because of a cardiac event. In contrast to this, our approach only assumes that a patient is conceptually at risk of experiencing any of these events, provided that none of these events has happened so far.

## Results

### General

We studied ten different scenarios, which are tabulated in Table 1 and cover all effect categories discussed earlier. Scenarios 1-5 have *β*_1 _similar to the actual study result [14], and scenarios 6-10 have *β*_1 _similar to the planning figures [21]. Table 1 also displays the use of different baseline hazards. For each of the scenarios, 1000 simulation runs were considered with 500 individuals in the placebo group and 500 individuals in the treatment group.

**Table 1:** Scenarios in the simulation study

			Transformation of	
Scenario	*β*_1_	*β*_2_		
1	-0.1	0	none	none
2	-0.1	0	*x *↦*x*^1/4^	none
3	-0.1	0.3	none	none
4	-0.1	0.3	none	*x *↦*x*^2^
5	-0.1	-0.3	*x *↦*x*^2^	*x *↦*x*^1/4^

6	-0.3	0	none	none
7	-0.3	0	*x *↦*x*^1/4^	none
8	-0.3	0.3	none	none
9	-0.3	0.3	none	*x *↦*x*^2^
10	-0.3	-0.3	*x *↦*x*^2^	*x *↦*x*^1/4^

Scenarios in the simulation study. Scenarios 1-5 have *β*_1 _similar to the actual study result, scenarios 6-10 have *β*_1 _similar to the planning figures.

Table 2 presents, for each scenario and based on Cox analyses of the CSHs, the mean log CSH ratios  and , empirical 95% confidence intervals, the coverage probabilities of the Wald-type confidence intervals for the estimated regression coefficients, and the empirical power.

**Table 2:** Simulation results

		Interest				Competing		
**Scenario**		**95% CI**	**Coverage probability**	**Empirical power**		**95% CI**	**Coverage probability**	**Empirical power**

1	-0.1	-0.32; 0.11	94.1	17.9	0	-0.27; 0.29	94.6	5.4
2	-0.11	-0.27; 0.06	95.9	24.3	0.01	-0.33; 0.35	95.1	4.9
3	-0.1	-0.31; 0.11	94.9	15.1	0.3	0.03; 0.56	94.5	64.5
4	-0.1	-0.3; 0.09	94.8	16.4	0.3	-0.09; 0.73	96.2	27
5	-0.1	-0.38; 0.21	96.1	11.1	-0.31	-0.5; -0.12	93.7	91.4

6	-0.3	-0.53; -0.09	94.1	78.8	0	-0.26; 0.27	95	5
7	-0.32	-0.5; -0.15	94.5	95.1	0	-0.32; 0.34	95.4	4.6
8	-0.29	-0.51; -0.09	95.7	75.9	0.29	0.04; 0.56	94.9	59.2
9	-0.3	-0.48; -0.09	95.5	81.4	0.31	-0.11; 0.78	94.5	26.9
10	-0.3	-0.61; 0.03	94.4	46.9	-0.32	-0.49; -0.14	95.5	92.1

Simulation results. For each scenario and for each competing risk are presented the averaged log hazard-ratios, empirical confidence intervals (CI), coverage probabilities and empirical power.

For Scenarios 1-5, we also plotted the true CIFs (solid black lines), the average of the Aalen-Johansen estimators (dashed black lines) and 300 randomly selected Aalen-Johansen estimators (grey lines). The reason to only plot a random subsample was to maintain a grey shading in the plots.

We collect some general observations. In the Figures, the true CIFs are visually hardly distinguishable from the average of the Aalen-Johansen estimators from each simulation study. This entails that the algorithmic point of view is appropriate: Simulation along this line yields data that are consistent with the original quantities. Similar statements hold for the average of the estimated regression coefficients and the coverage probability of their confidence intervals. The Figures also show that both regression coefficients and baseline hazards matter. E.g., keeping both regression coefficients, but changing baseline hazards alters the CIFs. Similarly, keeping both the CSH ratio of interest and the baseline hazards, but changing the competing CSH ratio alters the CIFs.

We also note that recovering the true CIFs implies that we would have also recovered the original cumulative hazards of Figure 2. This is so, because knowledge of all CIFs allows to derive all CSHs and vice versa.

### Scenarios 1 and 2: no effect on the competing CSH

Scenarios 1 and 2 are chosen with regression coefficients similar to the 4D study. Scenario 1 used the original cumulative baseline hazards  and scenario 2 used the transformed tuple . This transformation amplifies the hazard for the event of interest, because  except for the right tail of the time interval displayed in Figure 2.

**Figure 3:**
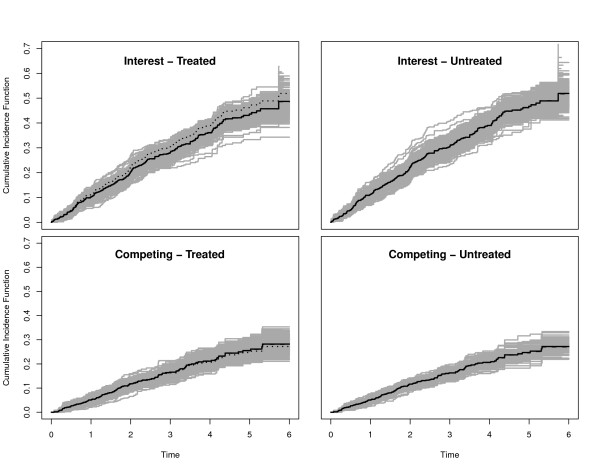
**Scenario 1 of Table 1**. Solid black lines are the true CIFs, solid grey lines are 300 randomly selected Aalen-Johansen estimators. The average of the Aalen-Johansen estimators is drawn as dashed black lines. Dotted lines in the left plots are the corresponding untreated CIFs.

**Figure 4:**
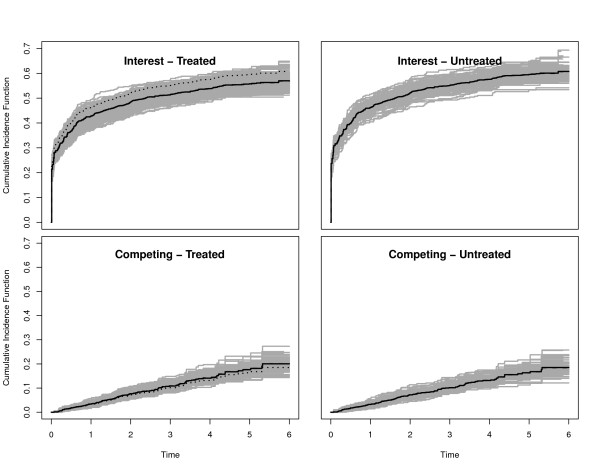
**Scenario 2 of Table 1**. Solid black lines are the true CIFs, solid grey lines are 300 randomly selected Aalen-Johansen estimators. The average of the Aalen-Johansen estimators is drawn as dashed black lines. Dotted lines in the left plots are the corresponding untreated CIFs.

Figures 3 and 4 illustrate that the CIF of interest is lower in the treatment group, as implied by *β*_1 _< 0 under the side condition *β*_2 _= 0. However, the difference is small, because the effect mirrored by *β*_1 _= -0.1 is rather moderate. The effect is somewhat amplified for the CIFs of interest when using  as a baseline CSH, because the transformation amplifies this hazard. The effect of a 'more important' baseline CSH for the event of interest is also reflected in the smaller empirical 95% confidence interval for the estimation of *β*_1 _and an increased empirical power. This is so, because increasing the baseline CSH of interest while not changing the competing baseline CSH will lead to more events of interest. In both scenarios, we find a slight increase of the competing CIF in the treatment group. Comparing both scenarios, one also finds that the overall magnitude of the competing CIF is reduced by amplifying the baseline CSH of interest. Finally, we note that the empirical power for the competing event approximately keeps the nominal level of 0.05.

### Scenarios 3 and 4: opposite treatment effects on the CSHs

**Figure 5:**
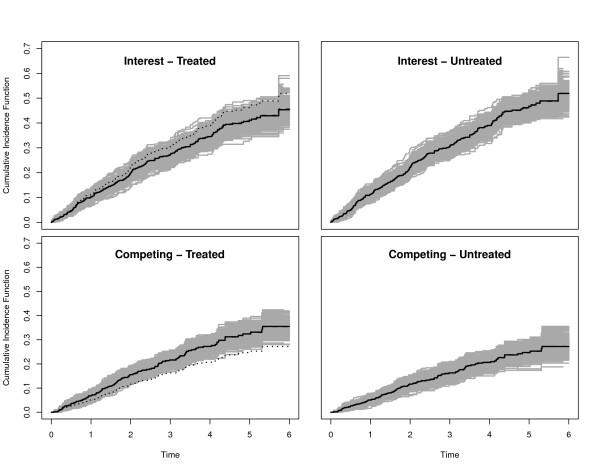
**Scenario 3 of Table 1**. Solid black lines are the true CIFs, solid grey lines are 300 randomly selected Aalen-Johansen estimators. The average of the Aalen-Johansen estimators is drawn as dashed black lines. Dotted lines in the left plots are the corresponding untreated CIFs.

**Figure 6:**
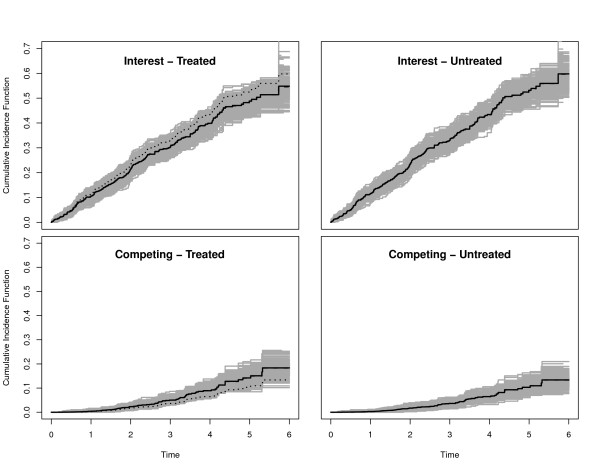
**Scenario 4 of Table 1**. Solid black lines are the true CIFs, solid grey lines are 300 randomly selected Aalen-Johansen estimators. The average of the Aalen-Johansen estimators is drawn as dashed black lines. Dotted lines in the left plots are the corresponding untreated CIFs.

Scenarios 3 and 4 are chosen with *β*_1 _similar to the 4D study, but *β*_2 _> 0 having an opposite effect. We deliberately chose |*β*_2_| > |*β*_1_| and, in scenario 4, transformed cumulative baseline hazards  to illustrate that the magnitude of a regression coefficient must be seen in connection with the magnitude of the corresponding baseline hazard. The transformation *x *↦ *x*^2 ^reduces the magnitude of the competing hazard, because . Figures 5 and 6 confirm a decreasing treatment effect on the CIF of interest and an increasing effect on the competing CIF. These effects are, however, diminished when using  as a baseline CSH, because the transformation reduces the magnitude of this hazard. This is, e.g., reflected in the empirical 95% confidence interval for the estimation of *β*_2_, which excludes 0 only in the case of untransformed baseline CSHs. In analogy to this, the empirical power is considerably reduced for the competing event in the transformed case.

### Scenario 5: unidirectional treatment effects on the CSHs

**Figure 7:**
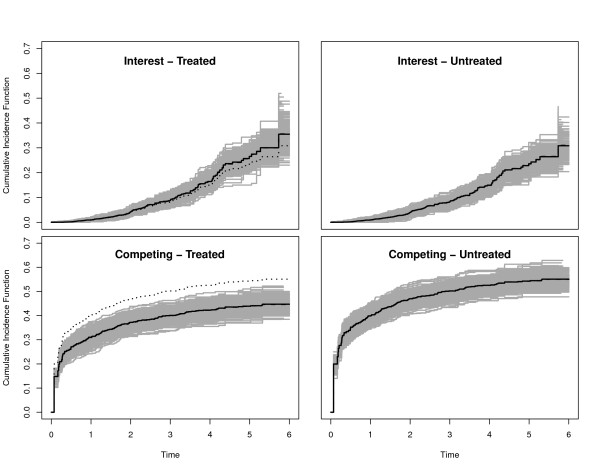
**Scenario 5 of Table 1**. Solid black lines are the true CIFs, solid grey lines are 300 randomly selected Aalen-Johansen estimators. The average of the Aalen-Johansen estimators is drawn as dashed black lines. Dotted lines in the left plots are the corresponding untreated CIFs.

We chose *β*_1 _= -0.1 as before and *β*_2 _= -0.3. Figure 7 displays the true CIFs with transformed cumulative baseline hazards . Because the transformation amplifies the competing CSH, but has an opposite effect on the CSH of interest, we see a slowed down increase of the CIF of interest in the control group as compared to the first two scenarios. There is a visible treatment effect on the competing CIF, which is reduced as compared to the control group. In contrast, the CIF of interest evolves at a comparable and low magnitude in both groups before eventually displaying larger probabilities for the treated. In fact, the CIFs of interest cross, i.e., the curve for the treated first runs below the one for the control group and then crosses. However, the early difference is hardly visible due to the overall low magnitude of the CIF of interest.

**Figure 8:**
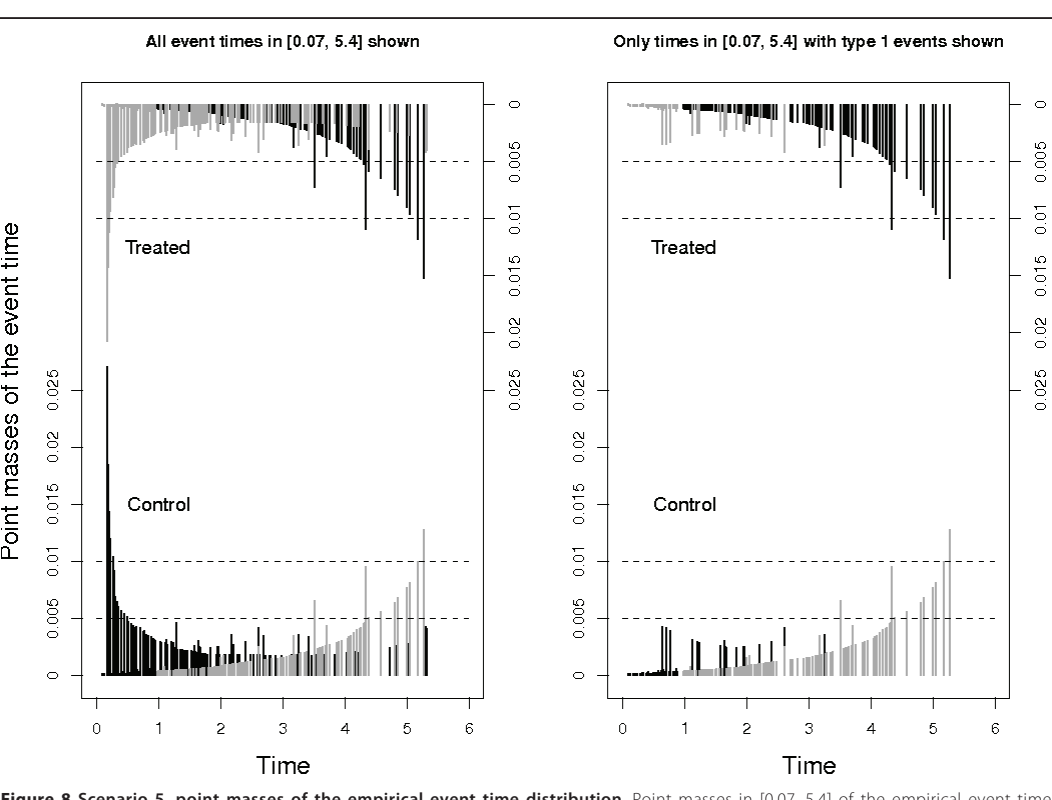
**Scenario 5, point masses of the empirical event time distribution**. Point masses in [0.07, 5.4] of the empirical event time distribution. The left plot shows all event times. The right plot is restricted to times with an event of interest. Bottom of the plots are for the control group with ordinate indicated on the left, top of the plots for the treatment group with ordinate indicated on the right. Black bars are larger than their corresponding bars in the other group, which then are grey.

The eventual difference between the CIFs of interest appears to contradict *β*_1 _< 0, but can well be understood from a simulation perspective as outlined in the *Overview of simulation scenarios*. This is illustrated in Figure 8. We discuss the left part of the Figure first. It corresponds to the first step of the simulation algorithm and illustrates that events of any type happen later in the treatment group, because both *β*_1 _and *β*_2 _are negative.

The plot shows the point masses of the empirical event time distribution for both treatment groups. The Figure has been restricted to [0.07, 5.4] in order to avoid the steep increases of the survival function towards the corners of the observed time interval. Black bars indicate larger point mass as compared to their corresponding bars in the other group. The corresponding bars indicating smaller point mass are grey. We also note that black bars do not really superimpose grey bars and vice versa in the Figure, although this impression is occasionally conveyed by the high density of bars. Figure 8 (left) clearly shows that probability mass is moved towards later event times in the treatment group. This is also reflected by the CIFs, which start to increase later for the treatment group, although this is hardly visible for the CIF of interest.

However, the left plot does not illustrate why the CIFs of interest cross. This is explained in the right plot. It corresponds to the second step of the simulation algorithm and illustrates that treatment shifts probability mass towards type 1 events in general and, more specifically, to *later *type 1 events as a consequence of *β*_2 _<*β*_1 _< 0, see the *Overview of simulation scenarios*.

To this end, note that in an actual data situation the empirical counterpart of step 2 of the simulation algorithm will often equal either 0 or 1, if there are no type 1 and type 2 events being observed at the same time. In our data example, both type 1 and type 2 events happened at only 18 out of 322 time points considered in Figure 8. Figure 8 (right) profits from the fact that the binomial probabilities are often degenerated and only shows those times with an observed type 1 event. If such a time is drawn, the event type is almost always determined to be of type 1.

The right plot illustrates two things: Firstly, black colour dominates the upper part of the plot, indicating that the event of interest is more likely to happen in the treatment group. Secondly, the colouring moves from grey to black for the treatment group and from black to grey in the control group. The interpretation is that, initially, event type 1 times are drawn with higher probability in the control group. The picture is being reversed as time proceeds, which leads to crossing CIFs, and eventually the cumulative proportion of type 1 events is larger in the treatment group. Note, however, that there is overall low probability mass on early type 1 event times, which implies that the CIFs of interest initially are hardly distinguishable and rather small.

An analogous plot of Figure 8 (right) for type 2 events shows that probability mass is almost uniformly reduced for type 2 event times by the treatment effect (figure not shown).

### Scenarios 6-10

Scenarios 6-10 repeated the previous investigations with a more pronounced treatment effect of *β*_1 _= -0.3. Results are reported in Table 2. The most striking difference to the results from scenarios 1-5 is an increased empirical power for events of type 1. The increased empirical power, however, does not only depend on *β*_1 _= -0.3, but on all aspects discussed above. E.g., both scenarios 6 and 7 have (*β*_1_, *β*_2_) = (-0.3, 0), but the cause-specific baseline hazard for type 1 events is amplified in scenario 7. This leads to scenario 7 having better empirical power than scenario 6. In contrast, power is substantially decreased for the situation studied in scenario 10.

## Discussion

This paper envisaged the CSHs as momentary forces of transition, which suggests an algorithmic perspective towards competing risks. 'Empirical simulations' worked as a proof of concept. The algorithmic perspective was used on the interplay between CSHs and CIFs.

The involved relationship between CSHs and CIFs has inspired a boost in methodological research on testing and direct modelling of the CIFs, e.g., [26-29]. Similar to our paper, a number of recent references have used simulation of competing risks data to investigate these methods. In particular, Gray's [26] test [5,30,31] and the Fine-Gray [27] model [22,32] have attracted attention. Both these exemplary references and the present paper found a subtle interplay between different CSH constellations and subsequent impact on the CIFs.

The difference to the present paper is that a typical simulation study will put the simulation algorithm aside as only a computational tool, once the data have been generated. Thus, one will typically specify the CSHs, use some simulation algorithm [13] for data generation and analyse the data with the methodology at hand. Then, the CSH specifications and the results of the data analyses will be compared. In contrast to this, we have advocated to use the simulation algorithm itself as an operational tool for interpretation. In this context, it is worthwhile to note that the algorithm of our paper does not experience a number of problems which come with the common latent failure time model. E.g., the problem of dependence of the latent times has motivated to include different dependence structures in some simulation studies. There is no such problem in our set-up, which therefore facilitates interpretation.

We discuss practical consequences next. To begin, it is interesting to revisit the results of the 4D study in the light of the simulation algorithm. As stated earlier, the original study finding was a CSH ratio of 0.92 for the event of interest and essentially no effect on the competing CSH. The competing CSH ratio was, of course, not exactly equal to 1.00, but it displayed a slight reduction. Because the treatment effect on the CSH of interest was moderate, and because the Nelson-Aalen estimators of the cumulative CSHs were not exactly proportional between treatment groups, the Aalen-Johansen estimators of the CIFs of interest display a somewhat subtle relationship in the original report. (Figure three in [14], not reproduced here.) E.g., the CIFs cross before displaying a moderate benefit for the treatment group. However, the difference between the CIFs is slight before crossing and must therefore not be overinterpreted. As a consequence, we believe that interpretation of the competing risks situation at hand is well guided by the idealised situation of the simulation scenario 1.

Next, we reiterate that it is crucial that all CSHs are analysed. In the Backgroung Section, we noted that this is often not the case in clinical research. We also illustrated that a comprehensive analysis should not be restricted to hazard ratios only, but that ideally the cause-specific baseline hazards will be considered, too. The bottom line is that the interpretation of the CSH ratio of interest depends both on the baseline CSH of interest, the competing CSH ratio and the competing baseline CSH. In particular, a missing analysis of the competing CSH may have seriously misleading consequences.

An important issue in this context is that of graphically presenting results. A popular and adequate choice are plots of the CIFs, which should, in particular, be plotted for all event types, if all competing risks are harmful. However, it was also illustrated that the connection between these plots and CSH analyses is not straightforward, such that further graphical tools would be helpful. The most obvious choice is to also show the estimated cumulative CSHs as in Figure 2. This should be done much more often. The reason is that the CSHs regulate the stochastic behaviour of the competing risks process as explained in the Methods Section and as illustrated in the Results Section.

In addition, plots such as Figure 8 which highlight the simulation perspective can be useful. The interested reader is also referred to 'vertical modelling' [33] and multistate incidence rate graphics [34]. We also note that 'vertical modelling' aims at modelling the binomial probabilities in step 2 of the simulation algorithm as a smooth curve. This approach is appropriate when taking the simulation algorithm as a starting point to model competing risks data. We reiterate that our aim has been different in that we took the simulation perspective as an operational tool to interpret the standard CSH analyses.

We return to the key fact that all CSHs should be analysed in the presence of competing risks, and that this is often not accounted for in clinical research. These issues raise the question of planning competing risks studies, see [35] for a recent review. If the aim is to compare CSHs, a typical assumption made during the planning phase of the study is that of constant or piecewise constant hazards, e.g., [21,36,37]. In addition, it is often assumed that the treatment does not affect the competing CSH, see [35]. In practice, it may be difficult to find an adequate closed form for time-dependent CSHs. Sample size calculations may become quite formidable, if the planned analysis is more complex than testing the CSH of interest only. Whatever the planned statistical experiment is, we note that our empirical simulation approach provides for a general tool to study empirical power and, hence, to decide on sample size, if data of a control group - or of patients similar to the anticipated control group - are available. This is also illustrated in Table 2. Interestingly, Figure 2 suggests that assuming constant CSHs might be a reasonable assumption for the present control group, but it should be pointed out that the simulation approach does not rely on such an assumption. It could be applied without further ado, if the CSHs show a pronounced time-dependency. In particular, one would not need to specify a closed form for the time-dependent CSHs. In closing, we mention that, while we have focused on the Cox model as the major tool to analyse CSHs, other models such as Aalen's additive model may be used for CSHs, too; see [38] for a recent textbook treatment. The simulation perspective of this paper may then applied to results from other CSH models, too.

## Conclusions

This paper suggests an algorithmic or simulation point of view for the interpretation of competing risks analyses. This point of view follows the construction of competing risks data based on the CSHs, envisaging the hazards as momentary forces of transition. Concerns on identifiability and plausibility that are common in the latent failure time context do not arise.

Simulation studies based on the empirical probability measure of a real data analysis served as a proof of concept. The simulation point of view was found to be adequate in that it recovered the original empirical law. Manipulating baseline hazards and treatment effects highlighted different aspects of a competing risks analysis.

All CSHs should be analysed, including the cause-specific baseline hazards. 'Empirical simulations' also provide a flexible tool for study planning in the presence of competing risks.

## Competing interests

The authors declare that they have no competing interests.

## Authors' contributions

AA, MS and JB conceived the study. AA carried out the implementation. JB and AA drafted the first version of the manuscript. All authors contributed to the writing and approved the final version.

## Pre-publication history

The pre-publication history for this paper can be accessed here:

http://www.biomedcentral.com/1471-2288/11/86/prepub
